# A Multiaxial Rehabilitation Programme for Workers with COVID-19 Sequelae Using a Conventional and Technological-Robotic Approach: The Proposal of INAIL and Fondazione Don Carlo Gnocchi

**DOI:** 10.3390/healthcare11111593

**Published:** 2023-05-30

**Authors:** Irene Aprile, Lucia Bramante, Chiara La Russa, Marco Germanotta, Valeria Teresa Barletta, Francesca Falchini, Lorenzo Brambilla, Eugenio Guglielmelli, Patrizio Rossi

**Affiliations:** 1 IRCCS Fondazione Don Carlo Gnocchi, 50143 Florence, Italy; iaprile@dongnocchi.it (I.A.); vbarletta@dongnocchi.it (V.T.B.); ffalchini@dongnocchi.it (F.F.); lbrambilla@dongnocchi.it (L.B.); 2 Central Medical Department, National Institute for Insurance against Accidents at Work (INAIL), 00144 Rome, Italy; l.bramante@inail.it (L.B.); c.larussa@inail.it (C.L.R.); pat.rossi@inail.it (P.R.); 3 IRCCS Fondazione Don Carlo Gnocchi, 20148 Milan, Italy; eguglielmelli@dongnocchi.it; 4 Department of Engineering, Università Campus Bio-Medico di Roma, Via Álvaro del Portillo, 21, 00128 Roma, Italy

**Keywords:** long COVID-19, rehabilitation, workers, robotics, telerehabilitation

## Abstract

The COVID-19 sequelae have been shown to affect respiratory and cardiological functions as well as neuro-psychological functions, and, in some cases, metabolic/nutritional aspects. The Italian National Institute for Insurance against Accidents at Work (Istituto Nazionale Assicurazione Infortuni sul Lavoro, INAIL) recorded that, until December 2022, 315,055 workers were affected by COVID-19; therefore, there is a need to identify an effective approach to treat such patients. Robotic and technological devices could be integrated into the rehabilitation programme of people with long COVID conditions. A review of the literature showed that telerehabilitation may improve functional capacity, dyspnoea, performance, and quality of life in these patients, but no studies were found evaluating the effects of robot-mediated therapy or virtual reality systems. Considering the above, Fondazione Don Carlo Gnocchi and INAIL propose a multi-axial rehabilitation for workers with COVID-19 sequelae. To accomplish this goal, the two institutions merged the epidemiological information gathered by INAIL, the expertise in robotic and technological rehabilitation of Fondazione Don Carlo Gnocchi, and the literature review. Our proposal aims to facilitate a multi-axial rehabilitation approach customized to meet the unique needs of each individual, with a particular emphasis on utilizing advanced technologies to address the current and future challenges of patient care.

## 1. Introduction

On 11 February 2020, the WHO announced the name of the respiratory disease caused by the new coronavirus: COVID-19. This novel coronavirus disease, induced by the virus SARS-CoV-2, originated in Wuhan (China) and spread worldwide, with 757,264,511 confirmed cases and 6,850,594 deaths (WHO update on 21 February 2023). Since the start of the COVID-19 outbreak in China, and its subsequent global spread, Italy has been one of the first countries to be affected, and the number of recorded cases has been among the highest in the world: after 3 years from the beginning of the pandemic, 25,547,414 confirmed cases and 187,850 deaths (Italian Ministry of Health, updated on 16 February 2023) were reported.

On 9 March 2020, in response to the increase in COVID-19 cases in the country, the Italian government decreed urgent measures promoting social distancing to limit the spread of the virus. From 11 March 2020, all non-essential activities, such as social interaction, sporting, retail, and recreational activities, were suspended and, when possible, most organisations had to introduce remote work: citizens were forced to stay at home and travelling was drastically reduced. People’s freedom to move was limited to urgent health circumstances or working in healthcare. A similar lockdown was never experienced before in Italy. Due to the rapid spread of the virus, China first and later Italy, went into a full lockdown, while other countries used social distancing as the principal strategy to limit the number of new cases [1].

After the dramatic management of the acute phase of COVID-19 and the need to contain the virus diffusion, through rapid and extensive preventive actions, such as lockdown, the mandatory use of face masks, and later vaccines, it is now crucial to manage COVID-19 sequelae [2]. There is strong evidence that the disease can become chronic or leave sequelae, the duration and reversibility of which are not already completely known. It describes itself as a “long COVID” disease, characterised by multiple symptoms occurring after the recovery from the acute phase, including fatigue, dyspnoea, chest pain, muscle and joint pain, palpitations, the persistence of anosmia and dysgeusia, hair loss, memory and attention deficit, anxiety, depression, vegetative disorders, and sleep disturbances [3,4].

Several factors that influence the course of the acute phase of the disease, such as pre-comorbidities, age, sex, gender, and body weight, could also play a role in long COVID. Indeed, assumptions were made about the existence of an alternative form of COVID-19 infection that affects the cells of the nervous system and develops in genetically predisposed individuals, mostly in women [5]. In addition, multi-organ involvement and dysfunction, prolonged bed confinement in more complicated courses, sedation, and mechanical ventilation are among the possible contributing causes of long COVID [6]. 

Rehabilitation is often necessary for individuals experiencing long COVID symptoms to improve their pulmonary [7], physical [8], and cognitive functions [9] and enhance their overall quality of life [10]. Over the years, advancements in robotics and technology have revolutionized the field of rehabilitation, providing patients with new and innovative treatment options. Robots, for instance, can be used to perform repetitive tasks or provide assistance with mobility, which is especially beneficial for patients with physical disabilities or limitations [11,12,13,14]. Technology such as virtual reality and gamification can also be used to create engaging rehabilitation programmes that can improve patient motivation and adherence to therapy. By using robotics and technology, clinicians can tailor rehabilitation programmes to the specific needs of each patient, helping them to improve their physical and cognitive function and enhance their overall quality of life.

The paper aims to propose a rehabilitation treatment protocol for workers affected by long COVID, by using new technologies and robotic rehabilitation. The epidemiological information gathered by the INAIL Institute, the Fondazione Don Carlo Gnocchi’s expertise in robotic and technological rehabilitation, and the literature review, are all considered in this regard.

## 2. Long COVID or Post-COVID Syndrome

According to several reports, 86% of patients affected by COVID-19 report at least one symptom associated with a long COVID diagnosis after follow-up, regardless of COVID-19 severity [15]. The most common symptoms of long COVID are hair loss (74.6%), headache (73.7%), depression (67.2%), anxiety (66.7%), joint pain (64%), and fatigue (58.8%) and are significantly more frequent in females than in males [16,17]. Moreover, anosmia, dysgeusia, sleep disturbances, and neuropathic and nociceptive pain can characterize the chronic stage of COVID-19.

For this type of chronic disease, there is increasing evidence for the need for a rehabilitation programme that seems to be more effective if implemented as early as possible [18,19,20]. Due to the complexity of patients with COVID-19 sequelae, rehabilitation in these cases should be tailored to the patient’s condition. The COVID-19 sequelae can affect respiratory and cardiological functions, neurological functions including psychological ones, and, in some cases, metabolic/nutritional aspects [21].

### 2.1. Respiratory Sequelae

A meta-analysis by Torres et al. [22] established decreased carbon monoxide diffusing capacity (DLCO) as the most common short- to medium-term respiratory function abnormality, occurring in 39% of hospitalised patients. Secondly, patients develop 15% of restrictive and 7% of obstructive patterns. The sustained sequelae in respiratory function are compatible with a restrictive pattern secondary to interstitial abnormalities [23]. Several studies have looked at functional capacity after COVID-19, especially the six-minute walk test (6MWT). Daher et al. [24] reported that 79% of patients, 30 days after discharge, had walking distances below their predicted values, of which 46% had values even below age-adjusted lower normal limits. Three-month values from another study [25] reveal that 22% of patients had a 6MWT < 80% as predicted; 16% of the patients desaturated, showing an association with a decrease in DLCO. The limitation of patients’ physical and functional capacities can be an aggravating factor for their quality of life. Reduced quality of life persists at six months [26], with greater severity and prevalence for those patients who were in critical condition during the acute phase, presenting more problems of mobility, pain/discomfort, and anxiety/depression.

### 2.2. Cardiological Sequelae

Post-acute COVID-19 syndrome (PACS) describes the clinical condition of some SARS-CoV-2-infected patients in which a wide range of signs and symptoms persist for several months after the acute phase of the disease. Cardiovascular symptoms including chest pain, dyspnoea, elevated blood pressure, palpitations, inappropriate tachycardia, fatigue, and exercise intolerance are common in this condition. Some infected patients develop cardiovascular diseases such as myocarditis, pericarditis, new or worsening myocardial ischemia due to obstructive coronary artery disease, microvascular dysfunction, stress cardiomyopathy, thromboembolism, cardiovascular sequelae of pulmonary disease, arrhythmias, while others have cardiovascular symptoms without objective evidence of cardiovascular abnormalities [27].

Concerning cardiac manifestations, it was initially believed that the frequency of cardiac involvement resulting from SARS-CoV-2 disease was directly associated with the severity of the clinical course of the pathology and the presence of comorbidities. Huang et al. [28] in recovered subjects who presented with cardiac symptoms during the acute phase revealed abnormal findings on cardiac magnetic resonance imaging (CMR) in 58% of patients 50 days after the onset of symptoms, notably decreased right ventricular ejection fraction and myocardial oedema suggestive of myocarditis and fibrosis. However, more recent CMR studies of individuals who recovered from COVID-19 have shown a high rate of cardiac involvement despite an asymptomatic or benign course of the disease [29,30].

### 2.3. Neurological Sequelae

Neurological symptoms and manifestations resulting from COVID-19 can occur before, during, or even after respiratory involvement [31]. The neurological sequelae after 6 months from COVID-19 are usually due to neuropathy, plexopathy, radiculopathy and/or myopathy (mainly derived from intensive care unit (ICU)), Guillain-Barré syndrome (GBS), cerebrovascular events (haemorrhagic or ischemic stroke), and encephalitis. Thus, sensory symptoms alone or in combination with paraparesis or tetraparesis, ataxia, and generalised areflexia can be observed in patients with sequelae involving the peripheral nervous system [23]. Hemiparesis, aphasia, cognitive function impairment (memory, attention, and executive functions), and behavioural disorders occur in patients with central nervous system sequelae. In terms of memory and attention, 18% claimed to have had memory impairment and 16% claimed to have experienced concentration impairment, 30 to 70 days after being discharged; the number was highest among patients admitted to the ICU [32]. Moreover, autonomic nervous system functions can be involved after COVID-19, late dysautonomia occurs in 2.5% of patients with post-COVID-19 conditions, with orthostatic hypotension, neurally-mediated syncope, and postural orthostatic tachycardia syndrome [33].

Pain can occur in patients with central and/or peripheral nervous system impairment, such as COVID-19 sequela. In particular, the prevalence of persistent pain after ICU has been estimated to range from 28% to 77% [34]. Persistent pain after ICU in patients with COVID-19 includes muscle pain related to joint contractures/muscle atrophy, or pain due to critical illness myopathy or polyneuropathy [35]. Specific procedures used to treat severe acute respiratory distress syndrome may also induce tissue/nerve injuries. In particular, peripheral nerve injury associated with prone positioning has been reported in 14.4% of COVID-19 survivors discharged to rehabilitation [36]. Other potential causes of neuropathic pain after ICU include complications from the supine position and/or traumatic procedures such as the placement of chest tubes or tracheostomy. Another potential mechanism for neuropathic pain after COVID-19 is the direct or indirect effect of the virus on the nervous system. Human coronaviruses are known to infect the peripheral or central nervous system through multiple mechanisms including cytokine secretions, general circulation of the virus, or direct invasion of the olfactory epithelium [37].

### 2.4. Psychological Sequelae

The SARS-CoV-2 pandemic has been a significant source of psychological distress. Fear of illness, death, uncertainty about the future, and social isolation resulting from the loss of educational and occupational activities are putting public mental health at risk [38].

Taquet et al. [39] discovered that patients with anxiety disorders were twice as likely to meet the criteria for a mental illness 90 days after diagnosis than patients with other non-COVID-19 pathologies. There was also an increase in mood problems and insomnia.

### 2.5. Metabolic Sequelae

Note that the diabetogenic potential of SARS-CoV-2 has been hypothesised, not only because of the targets used by the virus but also because of the inflammatory stress secondary to the disease. New-onset hyperglycaemia and acute metabolic decompensation from pre-existing diabetes mellitus (DM) are complications of COVID-19, especially among hospitalised patients [40].

## 3. Long COVID, Rehabilitation, and Technologies: Scientific Evidence

Preliminary data suggests that a personalised rehabilitation programme can be effective in patients with long COVID [41]. However, the risks of physical rehabilitation in these patients should be considered. In fact, some systematic reviews have underlined that rehabilitation may not be suitable after COVID-19 with severe lung or heart damage [41,42] or in patients with (a) postural orthostatic tachycardia syndrome, (b) myalgic encephalomyelitis, or (c) chronic fatigue syndrome [43]. On the other hand, there is evidence that in patients with involvement of the nervous system, early rehabilitation is effective [18].

Recently, a systematic review [44] investigated the effectiveness of rehabilitation interventions for subjects with post-acute COVID-19 syndrome, only focusing on RCTs. The review analysed 5 studies, including 512 participants. Overall, the results seem to suggest that rehabilitation improves dyspnoea, anxiety, and kinesiophobia; while results for pulmonary function were inconsistent. Finally, improvements were detected in muscle strength, walking capacity, sit-to-stand performance, and quality of life.

The COVID-19 era required a reorganisation of rehabilitation services based on current scientific knowledge and available technologies that can improve therapy in compliance with safety and security requirements. In particular, telerehabilitation represents an effective way to provide rehabilitation interventions, overcoming the limits imposed by the pandemic. According to the systematic review of Rivera et al. [45], telerehabilitation is an effective treatment for cognitive deficits in patients with neurological disorders, supporting the hypothesis that remote communication technologies are viable solutions for supporting healthcare interventions, as a quick and effective response to provide continuity of care and social connectedness during the COVID-19 pandemic.

With respect to the treatment of patients with COVID-19 sequelae, it is worth noting that, according to a recent systematic review of six randomised trials [46], telerehabilitation, compared to face-to-face treatments, usual care, or no treatment, may improve functional capacity, dyspnoea, performance, and physical components of quality of life without significantly increasing adverse events in people with COVID-19 and post-COVID-19 conditions.

In recent years, technology has spread to the rehabilitation area. Robotic rehabilitation allows for greater treatment intensity, more effective standardisation of the treatment protocols, and better objectivity in the measurement of the results. The possibility to verify the results after each session represents a stimulus for the patient and an important tool for physicians and physiotherapists in the planning of the therapeutic programme. Patients’ motivation is fuelled and maintained both by the sensory stimuli that support the robotic treatment and by the challenge to achieve even better results. Above all, robotics allows patients to perform movements and tasks that would otherwise be impossible, thus accelerating the recovery process in the most acute and potentially most important phases. Similarly, VR can provide patients with more sensory stimulation, a more immersive environment, and real-time feedback during specific motor tasks, reflecting motor learning and neuroplasticity. However, systematic reviews on the use of other technologies, such as robotics or VR, to restore physical and cognitive functions in patients after COVID infection were not retrieved. Therefore, we perform literature research on this topic. The primary objective was to determine whether these cutting-edge advancements have been effectively harnessed for addressing the unique challenges posed by long COVID. Specifically, PubMed and the Web of Science were used to perform the literature search. The electronic search was conducted in May 2022. Across referencing was used from each publication obtained via the electronic search to avoid missing key studies. The search strategy, combining relevant search terms with Boolean operators (OR/AND), is reported in Table 1.

**Figure 1:**
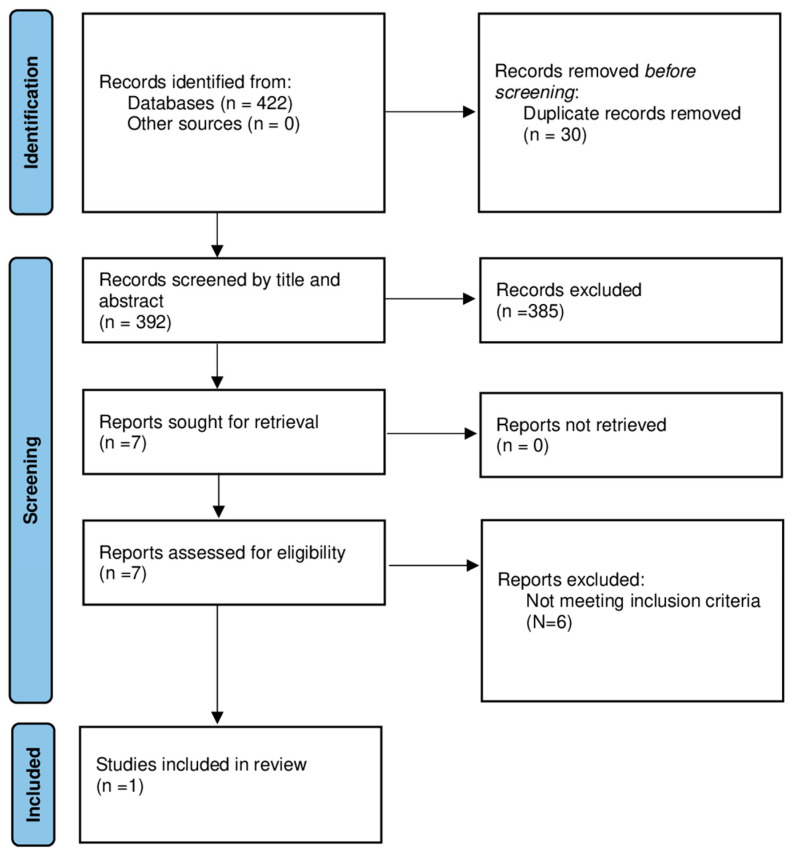
Flow chart.

We included RCT, non-randomized trial, or study protocol on patients with COVID sequalae reporting physical, cognitive, disability, or quality of life outcome measures. Figure 1 depicts the study selection procedure by displaying the number of studies that were retrieved, excluded, and included. We only found a protocol for developing, deploying, and evaluating digitally enabled remote, supported rehabilitation for people with long COVID-19 syndrome that included several validated patient-reported outcome measures (PROMs) [47]. According to our research, no studies evaluating the effects of robotic-based or VR on patients with long COVID syndrome were retrieved.

## 4. The INAIL Proposal for Long COVID Rehabilitation

The Italian National Institute for the Prevention of Accidents at Work (INAIL) is a state-managed insurance for workers, which provides compensation to insured subjects in case of work-related injuries and diseases.

In the current COVID-19 emergency, INAIL recognises COVID-19 as a work-related injury, and thus, it compensates the affected, in case of permanent outcomes.

In this exceptional pandemic, INAIL recorded 315,055 injuries from COVID-19, 891 of them with a fatal outcome. The last INAIL report emphasised that 68.4% of infections involved women while 31.6% involved men. The female component exceeds the male component in all regions except Campania, with female incidences of 49.3%.

The average age is 46 for both sexes and the median age is 48. In December 2022, the average age and the median age increased, and they are 48 and 50, respectively.

In Italy, COVID-19 involved approximately 88.5% of Italian workers and about 11.5% of foreigners, mostly from Romania (20.7%), Peru (12.3%), Albania (7.3%), Moldavia (4.3%), Switzerland (4.6%), and Ecuador (4.0%).

Regarding the territorial distribution of infections, data reveal that about 40.6% of the north-west population of Italy has been affected by COVID-19 (Lombardy first with 23.5%), 21.5% in the northeast (Veneto 10.7%), 16.8% in the centre (Lazio 8.4%), about 14.9% of the south (Campania 7.6%), and about 6.2% of the islands (Sicily 4.5%).

The most affected provinces were Milan (9.5%), Turin (6.7%), Rome (6.6%), Naples (4.6%), Brescia (3.1%), Genoa (3.2%), Treviso and Verona (2.1% each), Venezia (2.2%), Vicenza, Monza and Brianza (2.0% each), Florence and Varese (both with 1.9%), and Bologna (1.8%).

Healthcare professionals were the most affected with a rate of 37.5%; 82.3% of them being nurses, 16% socio-health workers, 9.4% doctors (internal medicine specialists and general practitioners), 5.4% social welfare workers, and 4.4% other non-specialised personnel in healthcare services (auxiliary, porters, stretcher bearer) [48].

In the healthcare sector, the virus contagion went through changes between 2020 and 2021: the infection reached its peak in the first half of 2020, and was at its lowest in the second half of 2020. COVID-19 contagion picked up again in the second part of 2021, reaching 2000 cases in December and five thousand in January 2022. In 2022, the infection increased with incidence levels very close to the most acute periods observed during the pandemic.

The remaining personnel involved include clerks in charge of checking documents and sorting and delivering mail (2.3%), cleaning service workers (1.9%), clerks in charge of branches and money movements (1.5%), vehicle drivers (1.2%), and clerks in the security, surveillance, and custody services (1.2%).

The end of mandatory smart working influenced the rates of virus contagion in other occupations. From February 2021 to the second half of the year, including the first month of 2022, the diffusion of infection increased, especially for certain occupations such as clerks in charge of documentary checks and mail delivery and sorting, primary school teachers, etc. [48].

By December 2022, the INAIL physician had evaluated approximately 2564 patients infected with COVID-19, of which 25% were evaluated without sequelae. According to the literature, the more common COVID-19 sequelae in workers involve the pulmonary function, psychological function, fatigue, neurological function, and cardiovascular function. Furthermore, 71% of workers show interstitial pneumonia and dyspnoea outcomes, and about 23% have psychic disorders such as post-traumatic stress disorder and adjustment disorder, with symptoms of anxiety and depression, and about 14% of the examined sample show sensory alterations of smell and taste and fatigue.

About 13% of workers show signs of neurological disorders, which include cognitive changes such as short-term memory deficits, deficits in focus and attention, and peripheral neurological conditions such as diffuse paraesthesia. Post-intensive critical neuropathy has been reported very rarely.

Lastly, 10% of workers experienced vascular consequences, such as thrombotic events that impacted the lungs, lower limbs, and brain tissues.

The reported data were retrieved by CarcliWeb, which is a web-based procedure that is utilized by medical doctors employed by INAIL when they take charge of patients who have sustained work-related injuries or illnesses. The specific records of patients with COVID-19 were imported into a Microsoft Excel database including the following data: age, sex, work activity, location and date of the event, degree of disability, and description of the reported pulmonary, psychological, neurological, or cardiovascular sequelae. In the current exceptional COVID-19 pandemic, and due to the high number of COVID-19 patients, INAIL plays a supporting role towards the National Health Service, in order to provide early and optimal rehabilitation treatment. Because of COVID-19 multi-organ sequelae, INAIL developed a multi-axial rehabilitation model that assists different rehabilitation settings to facilitate COVID-19 victims’ recovery.

Multi-axial rehabilitation (RMCo-19) [49] should include the following rehabilitation settings:

- Respiratory rehabilitation: based on personalised evaluation and treatment, which includes exercise training, education, techniques of bronchial clearance, and behavioural modification designed to improve the physical and psychological condition of people with respiratory diseases.
- Cardiac rehabilitation: based on healthy behaviour and education, lifestyle risk factor management, medical risk management, long-term strategies, and exercise training with ergometers at different frequencies and intensities aimed to improve cardiac function.
- Musculoskeletal rehabilitation: based on traditional and aquatic exercise training and/or technological and robotic solutions aimed at improving muscle strength, endurance, overall motor performance, and the gradual recovery/adaptation of daily life activities.
- Neuropsychological rehabilitation: based on cognitive-behavioural techniques aimed at improving higher functions and psychological aspects.

## 5. Fondazione Don Carlo Gnocchi for Long COVID Rehabilitation

**Figure 2:**
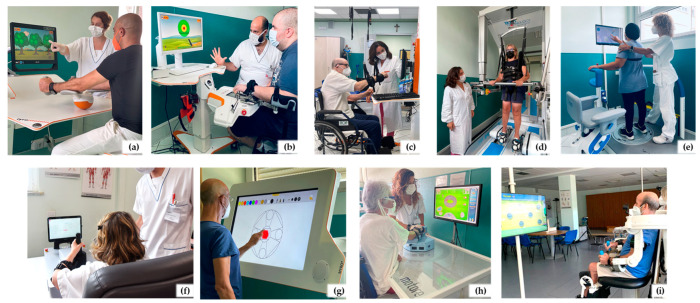
Examples of technological rehabilitation devices within the Fondazione Don Carlo Gnocchi Centres: sensor-based (**a**,**g**), robotic (**b**,**f**,**h**,**i**), and electromechanical (**c**) devices for upper limb rehabilitation; robotic devices for gait (**d**) and balance (**e**) rehabilitation.

Fondazione Don Carlo Gnocchi (FDG) is the largest private, non-profit organisation in the field of rehabilitation in Italy. It has 28 residential facilities, around 30 outpatient clinics, and in-depth experience in robotic and technological rehabilitation (Figure 2) [50,51].

**Figure 3:**
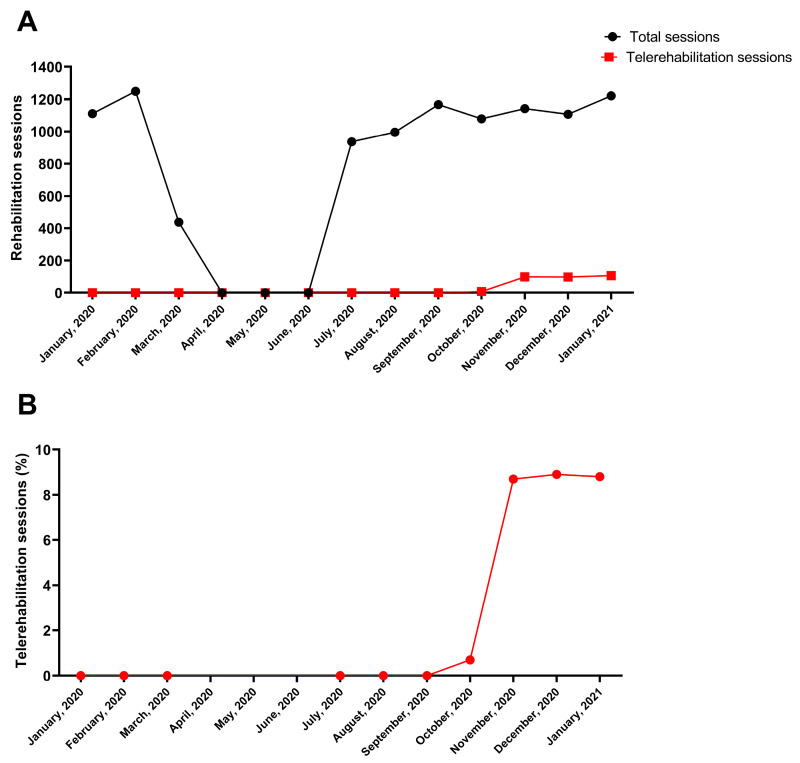
Outpatient rehabilitation sessions provided monthly by the centre Santa Maria della Provvidenza of the Fondazione Don Carlo Gnocchi in Rome, between January 2020 and January 2021: (**A**) number of rehabilitation sessions (total, in black, and with telerehabilitation services, in red); (**B**) percentages of sessions provided by telerehabilitation services.

During the pandemic, FDG continued to provide rehabilitation services to patients, particularly by utilising various technologies. In order to accomplish this, FDG acquired a specific CE-certified platform that gave it access to patients’ homes throughout the lockdown. Following a training period, telerehabilitation sessions based on real-time interactivity (synchronous interaction) now make up around 10% of outpatient rehabilitation sessions (Figure 3).

## 6. Integrated Multi-Axial Rehabilitation Protocol INAIL-FDG

**Figure 4:**
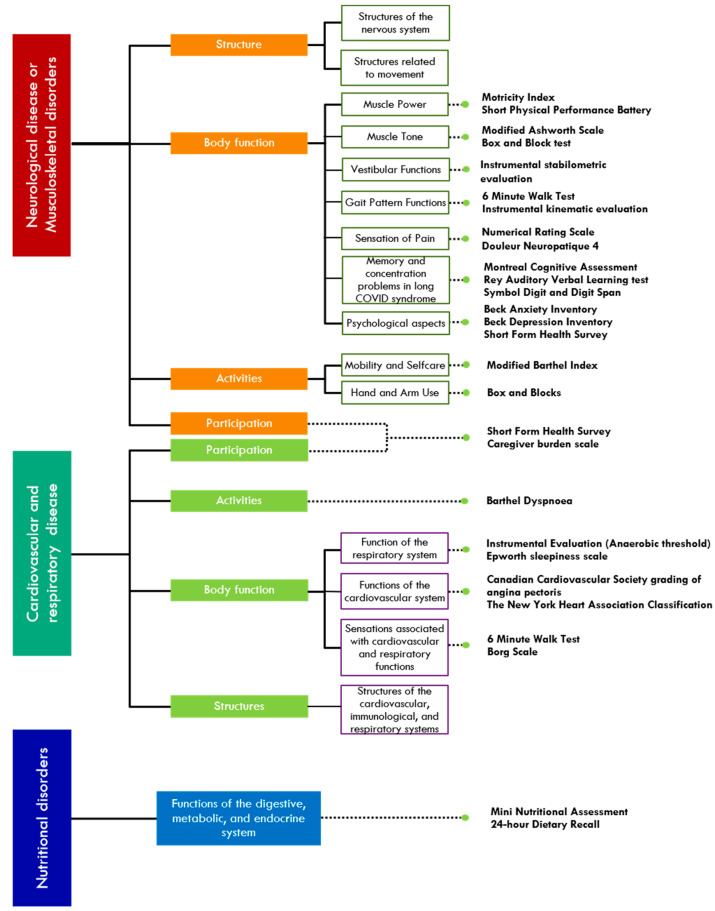
Proposed evaluation according to the International Classification of Functioning, Disability, and Health (ICF) [52].

In the last year, Fondazione Don Carlo Gnocchi has started a collaboration with INAIL to ensure multi-axial rehabilitation for COVID-19-affected workers integrated with technological and robotic devices. The protocol is characterised by a multiperspective approach, including respiratory and cardiological rehabilitation, neuromotor rehabilitation, cognitive rehabilitation, and psychological and nutritional treatment. The protocol takes advantage of the beneficial effects of robotics and technologies on sensorimotor and cognitive deficits [51], which have been widely demonstrated in neurological diseases and could be effectively used to treat the long-term effects of COVID-19 infection. FDG also proposed a set of validated outcome measures to evaluate the effects of rehabilitation, selected in light of the International Classification of Functioning, Disability, and Health (ICF) framework, to evaluate (a) structure; (b) body function (muscle power, sensation, pain); (b) activities (ability in daily living); and (c) participation (quality of life) (Figure 4).

### 6.1. Respiratory and Cardiological Rehabilitation

Respiratory function rehabilitation can be divided into two main categories: (a) rehabilitation of respiratory function and motor performance; and (b) bronchial disruption. The rehabilitation of respiratory function and motor performance involves motor training exercises aimed at improving the strength and endurance of the upper and lower limbs, and respiratory muscles [53,54,55,56]. Cardiological rehabilitation includes a personalised programme of aerobic exercise tailored to the patient’s exercise tolerance [57,58,59,60]. Simple interval training programmes on cycloergometers may be proposed for lower and upper limbs [61,62,63,64]; segmental exercises with elastic bands or small weights are advisable when possible to increase exertion. The programme’s purpose is to minimise deconditioning and sarcopenia, both factors that can lead to an increase in dyspnoea. In particular, the training programme is aimed at improving muscle oxidative capacity, the efficiency of contraction and cardiovascular function, kinetics, and oxygen consumption (VO_2_) in the recovery phase, and the reduction of the level of lactates produced during the exercise and the share of non-metabolic carbon dioxide (CO_2_) generated by the bicarbonate system to buffer its effects on pH.

Bronchial unblocking is an important element of rehabilitation, especially for patients with hypersecretion. The techniques used are: (a) the Active Cycle of Respiratory Techniques (ACBT), (b) the Total Slow Expiration with Open Glottis in Lateral Decubitus (ELTGOL) [65], (c) the Autogenic Drainage (AD), and (d) the Positive Expiratory Pressure (PEP Mask) [66], with the possible addition of oscillations (Flutter, Cornet, Acapella, etc.) [67]. Furthermore, mechanical instruments can be applied for intra- or extra-thoracic compression/oscillation (IPV, HFCC) in more complex cases or patients with neuromuscular difficulties [68,69]. A rehabilitation pathway should always be suggested for patients with diffuse interstitial illness (as per SARS-CoV-2 outcomes), as it increases exercise tolerance, albeit in relation to the severity of lung dysfunction and to the desaturation levels during exercise testing [70,71].

Finally, the continuation of such a rehabilitation programme, also through telerehabilitation and the telemonitoring of heart rate, body pressure, pulse oximetry, and hemoglobin, is recommended since exercise’s physiological effects decay if ad hoc maintenance programmes are not provided [56].

Therefore, we suggest using the following outcome indicators to assess the impact of respiratory and cardiovascular rehabilitation: Barthel Dyspnoea [72]; Instrumental Evaluation (Anaerobic threshold) and the Epworth sleepiness scale [73]; the Canadian Cardiovascular Society (CCS) grading of angina pectoris [74] and the New York Heart Association (NYHA) classification [75]; the Six-Minute Walking Test (6MWT) [63]; and the Borg Scale [76].

### 6.2. Neuromotor Rehabilitation

The focus of neuromotor rehabilitation in patients with SARS-CoV2 outcomes relies on the treatment of the central and/or peripheral nervous system. Neuromotor rehabilitation should be performed by the integration of conventional techniques with new advanced technological and robotic devices.

Conventional rehabilitation allows for functional recovery of the limbs and trunk, as well as the complex tasks that rely on them, such as walking, balance, coordination, and postural control, all with the goal of regaining autonomy in ADLs.

Conventional re-education protocols should promote:

- sensorimotor training to restore normal motor programmes, maintain joint mobility of the upper and lower limbs through passive and active assisted mobilisations, and control pain when present;
- programmes to increase muscle strength and endurance with incremental exercises appropriate to the patient’s performance, and programmes to recover segmental movement and then more complex motor functions;
- training from autonomies in safe postural transitions;
- postural control exercises;
- balance and gait recovery exercise;
- activities aimed at family and social reintegration.

Robotic rehabilitation grants treatment of both upper and lower limbs; they represent an innovative rehabilitation approach that allows:

- intensified treatment;
- objective measurement of patient’s achieved goals;
- personalised treatment;
- stimulation of neurocognitive as well as motor aspects [77].
- The robotic device can be:
- End-effector, where movements are generated from the most distal segment of the extremity and no alignment between patient-robot joints is required [78];
- Exoskeletons, which have a one-to-one correspondence between robots and human joints, and every single joint is guided along a pre-programmed trajectory [78]. They can support and assist complex motor functions of the upper and lower limbs.
- There are also advanced technological systems such as:
- sensor-based systems that allow for the control of a wide range of limb movements, but also of the whole body in space (trunk and gait control);
- electromechanical systems with body weight or a district relief system (e.g., upper limb).

Robotic rehabilitation approaches can be useful for post-COVID symptoms such as disabling balance disorders and insufficient postural control using technological/robotic platforms. Stabilometric and dynamic platforms can also be used to carry out evaluative tests of the patient’s condition before and after the rehabilitation course. Moreover, exoskeleton or end-effector robotic devices (that safely sustain the patient using a weight body support system), allowing more physiological gait training even in the presence of a significant lower limb strength deficit, can be utilised for gait training.

Some sensor-based and robotic devices have been found to be useful not only for inpatient rehabilitation, but also for home-based rehabilitation with caregivers (telerehabilitation and telerobotic programmes through telemonitoring). Through monitoring systems, the therapist evaluates the patient’s progress every day and adjusts the treatment to suit the patient’s needs. Furthermore, telerehabilitation systems also allow for the simultaneous treatment of patients in small groups.

We propose to evaluate the effects of neuromotor rehabilitation using the following outcome measures: the Motricity Index [79], to evaluate upper and lower limb strength; the Short Physical Performance Battery [80], to assess lower extremity functioning; the 6-min walk test [81], to assess endurance; the Box and block test [82], to assess gross manual dexterity; the Modified Ashworth Scale [83], to evaluate spasticity; the Modified Barthel Index [84], to assess disability; the Numeric Rating scale [85], to assess pain intensity; and the Douleur Neuropatique 4 [86], to identify neuropathic pain. Moreover, we suggest including an instrumental evaluation of both balance (using stabilometric platforms [87]) and gait (using optoelectronic systems [87], or inertial measurement units for remote assessment [88]).

### 6.3. Cognitive Rehabilitation

#### 6.3.1. Memory and Concentration Problems in Long COVID Syndrome

Long COVID’s cognitive deficits, including memory loss and moderate cognitive impairment, might last for months after recovery from the acute phase.

The most experienced symptoms are memory issues, multitasking deficits, processing speed reduction, and lack of concentration. These symptoms can prevent the return to normal activities of daily living. Therefore, early interventions aimed at limiting short- and long-term damage are important.

It is first necessary to conduct a neuropsychological assessment to highlight cognitive deficits and to characterise their impact on the individual, with the aim of guiding treatment. The assessment will be carried out through a cognitive screening test and, later, with specific tests for individual impaired areas.

Therapy will be trained according to the functions that are most affected on the assessment tests or experienced as more debilitating by the patient.

Cognitive training aims at reducing symptoms and will be based on two works conducted in parallel:

- targeted reinforcement of deficit skills through the administration of exercises of increasing difficulty (e.g., short-term memory training or attention training);
- adaptation of the patient’s behaviours and setting of new compensation strategies (e.g., for memory difficulties the use of tools such as calendars and alarm clocks; for concentration difficulties the avoidance of distracting situations and contexts, to do only one thing at a time);
- evaluation of the results obtained is mandatory at the end of the treatment.

The first interview and administration of standardised tests should be conducted in person; cognitive training could be conducted in telerehabilitation or mixed regimens.

To assess the effectiveness of the proposed intervention, we suggest using the following outcome measures: the Montreal Cognitive Assessment [89], to evaluate general cognitive impairment; the Digit Span [90], to assess working verbal memory; the Symbol Digit Modalities Test [91], to assess information processing speed (in terms of attentional capacity and working memory); and the Rey Auditory Verbal Learning test [92], to assess attention, memory, and learning ability in the auditory-verbal domain.

#### 6.3.2. Psychological Aspects

The lockdown has had an impact on the whole community, particularly in terms of family separation. This mostly affected those who were unable to spend the lockdown period with their family. This situation led to psychological distress for patients [93].

To overcome this distress, adequate psychological support for patients should be conceived, and “technological” solutions, such as video calls, can be used to maintain communication with patients also after dismission.

In this context, we suggest evaluating anxiety, using the Beck Anxiety Inventory [94], depression, using the Beck Depression Inventory [95], and the quality of life of patients, using the Short Form Health Survey (SF-36) [96]. In addition, we recommend evaluating the caregiver burden using the Caregiver Burden Scale [97].

#### 6.3.3. Nutritional Aspects

Significant weight loss (sarcopenia) is common in post-COVID patients with severe symptoms due to a long ICU stay. Nutritional problems need particular attention as they result in reduced exercise tolerance, susceptibility to infection, and consequently, to worse outcomes. Therefore, it is critical to employ nutritional status screening tools to identify people who are malnourished or at risk of malnutrition, and to implement procedures to monitor and regulate daily nutritional intake in these circumstances.

For this purpose, we propose the use of the Mini Nutritional Assessment [98] to grade the nutritional status of patients and the 24-h Dietary Recall [99] to assess individual diets.

## 7. Discussion

Scientific evidence shows that SARS-CoV-2 infection may affect health at different levels. Due to the COVID-19 multi-organ sequelae, an exceptional number of workers underwent medical complications and lost their ability to work and conduct a normal life.

In this scenario, INAIL plays a crucial role in supporting Italian Public Healthcare to guarantee optimal multi-axial rehabilitation assistance in terms of the appropriateness and timeliness of the rehabilitation intervention.

As an institution created to protect and support workers’ wellbeing integrity, INAIL provides multi-axial rehabilitation assistance for those whose health has been affected by the occurrence of the pandemic. These services are dispensed in specific rehabilitation institutes such as the centres of Fondazione Don Carlo Gnocchi. These centres provide professional expertise, specialised structures, and up-to-date technologies, such as robotic devices, to rehabilitate different types of disabilities.

An optimal multi-axial rehabilitation approach entails stepping in at the earliest stages of the pathological process to promote healing and improve clinical conditions, selecting the most effective treatments according to scientific evidence and good practice. This requires including multiple district interventions and contemplating the possibility of repeated treatments (deferred rehabilitation).

As we described in this proposal, both conventional and technological treatments should be applied. Technological-robotic rehabilitation has been proven to be a promising tool for both physical therapy and remote interventions (telerehabilitation).

Telerehabilitation has expanded its applications in the last 25 years due to the development of new computer science technologies [100]. During the COVID-19 pandemic, it was a precious resource for patients and caregivers. After physical interactions were restored, telerehabilitation, and telemedicine in general, kept catching on in medical practice. As of today, several applications for remote assistance have been identified, which promise to provide beneficial innovation to patient care in a world that is quickly changing [101]. Recently, some experiences have emerged in the field, including those involving the utilization of robotic devices specifically designed for home-based application [102]. These experiences have garnered attention due to their potential to revolutionize the way in which individuals can receive therapeutic interventions and rehabilitation services within their own homes.

Whatever type of treatment we intend to apply, reintegrating workers with long COVID sequelae in their jobs, families, and social activities will undoubtedly have a positive impact on their quality of life and will reduce the costs related to days of absence from work and medical complications. In order to ascertain the efficacy of the proposed treatment in individuals experiencing long COVID outcomes, it is imperative that experimental studies be undertaken. These investigations should involve rigorous clinical trials or observational studies, employing robust methodologies and a sufficiently large sample size. By carefully monitoring the response of patients to the treatment, including its impact on various symptom domains, functional outcomes, and quality of life measures, researchers can acquire valuable evidence regarding its effectiveness. Additionally, long-term follow-up assessments should be conducted to evaluate the durability and sustainability of the treatment outcomes over an extended period. The findings derived from such studies will not only contribute to the existing body of knowledge, but also provide crucial insights for healthcare professionals and policymakers when determining the most appropriate therapeutic interventions for patients suffering from long COVID.

## 8. Conclusions

The COVID pandemic has been a hard test for Italian healthcare professionals, patients, and their caregivers in terms of the management of the medical emergency and because of the human and social costs that it caused to the country.

Aside from providing adequate assistance to the affected workers, our proposal intends to promote a multi-axial rehabilitation approach that is effectively tailored to individual needs, taking into special account the availability of advanced technologies and their application to the current and future challenges of patient care.

## Figures and Tables

**Table 1:** Search strategy.

**Pubmed**	(Rehab*[tiab]) AND (COVID-19[tiab] OR “COVID 19”[tiab] OR “COVID”[tiab] OR long-covid[tiab] OR COVID-2019[tiab] OR SARS-CoV-2[tiab] OR 2019-nCoV[tiab] OR 2019-SARS-CoV-2[tiab]) AND (robot*[tiab] OR “robot assisted”[tiab] OR “robot-assisted”[tiab] OR exoskelet*[tiab] OR “end effector”[tiab] OR end-effector[tiab] OR electromechani*[tiab] OR electro-mechani*[tiab] OR “Virtual Reality” [tiab] OR VR[tiab] OR Kinect[tiab] OR “wii” [tiab] OR technology[tiab] OR “augmented reality” [tiab])
**Scopus**	TITLE-ABS-KEY (rehab*) AND TITLE-ABS-KEY (COVID-19 OR “COVID 19” OR long-covid OR covid OR COVID-2019 OR SARS-CoV-2 OR 2019-ncov OR 2019-SARS-CoV-2) AND TITLE-ABS-KEY (robot* OR “robot assisted” OR “robot-assisted” OR exoskelet* OR “end effector” OR end-effector OR electromechani* OR electro-mechani* OR “Virtual Reality” OR vr OR kinect OR “wii” OR technology OR “augmented reality”)

## Data Availability

All relevant data are available within the article.
